# Case Report: Infiltrative multifocal meningioangiomatosis affecting the spinal cord of a young Labrador Retriever

**DOI:** 10.3389/fvets.2025.1580306

**Published:** 2025-06-10

**Authors:** Hana Gunovska, Sara Degl’Innocenti, Mike Targett, Ines Carrera, Sergio A. Gomes

**Affiliations:** ^1^Dovecote Veterinary Referrals, Derby, United Kingdom; ^2^School of Veterinary Medicine and Science, University of Nottingham, Nottingham, United Kingdom; ^3^VetOracle, Norfolk, United Kingdom

**Keywords:** neoplasia, myelopathy, hamartoma, MRI, neuroimaging

## Abstract

Meningioangiomatosis is a rare leptomeningeal and meningovascular proliferative disorder of the central nervous system. Predilection site in dogs is the brainstem, with scarce reports of unifocal spinal meningioangiomatosis. This is the first case report of multifocal spinal meningioangiomatosis affecting the cervical, thoracic, and lumbar regions. A 2-year-old male Labrador Retriever presented for progressive ambulatory paraparesis. Vertebral column MRI revealed two presumed intramedullary space-occupying lesions, at T8–9 (large, occupying most of the spinal cord) and L3–4 (smaller, confined to grey matter); both T2-weighted hyperintense, T1-weighted isointense with contrast enhancement. Surgical exploration with durotomy revealed a unresectable well-defined mass distinct from the parenchyma of the spinal cord. Following neurological deterioration, euthanasia was performed. Multifocal to coalescing leptomeningeal and meningovascular proliferations consistent with were found on necropsy, many non-visible on MRI. This case highlights a possible differential diagnosis for multifocal spinal intramedullary masses in young dogs.

## 1 Introduction

Meningioangiomatosis (MA) is a rare, complex, and heterogenous proliferative disorder of the central nervous system (CNS). It is described as a non-neoplastic, focal lesion arising within the leptomeninges and compressing and/or infiltrating the underlying neural parenchyma, characterized by leptomeningeal and meningovascular proliferation (1). The etiology and nature of MA remain elusive, with proposed etiologies including developmental (hamartomatous), dysplastic, or reactive processes, rather than neoplastic (1).

MA is reported as a rare condition in man (2), with 15 reported cases in dogs (1, 3–13) and single case reports in cat (14), horse (15), cow (16), and mouse (16). There is an overrepresentation of Labrador Retrievers in reported cases (4/15), affecting more frequently young dogs (1, 3–13). The vast majority of canine MA cases have been reported within the brain (12/15), predominantly in the brainstem. It is characterized histologically by either single, focal, or multiple lesions in close proximity. Spinal cord MA has been described only in three cases, as a single lesion affecting the level of C4–C6 and the level of T13 and L1 vertebrae (8, 11).

We describe the first case report of MA with extensive infiltrative multifocal localization within the spinal cord affecting the cervicothoracic, thoracic, thoracolumbar, and lumbar regions, with some lesions not identifiable on MRI.

## 2 Case description

A 2-year-old male entire Labrador Retriever was presented to the Neurology and Neurosurgery department at Dovecote Veterinary Referrals for a 3-week history of progressive pelvic limb dysfunction.

General clinical examination was largely unremarkable. Neurological examination revealed ambulatory paraparesis with proprioceptive ataxia, with intact segmental spinal reflexes. The remainder of the neurological examination was normal. A neurolocalization to the T3–L3 spinal cord segments was made.

The dog was anesthetized, and magnetic resonance imaging (MRI) of the whole vertebral column and brain was performed using a 1.5 Tesla magnetic resonance scanner (Siemens MAGNETOM Sempra, Erlanger, Germany). The following sequences were obtained: T2-weighted (T2W) in the sagittal and transverse planes; short T1 inversion recovery (STIR) in dorsal and transverse planes; T1-weighted (T1W) in transverse planes pre- and post-contrast, and a modified fast 3D T1 gradient-echo sequence thin-slice volumetric interpolated breath-hold examination post-contrast.

**Figure 1:**
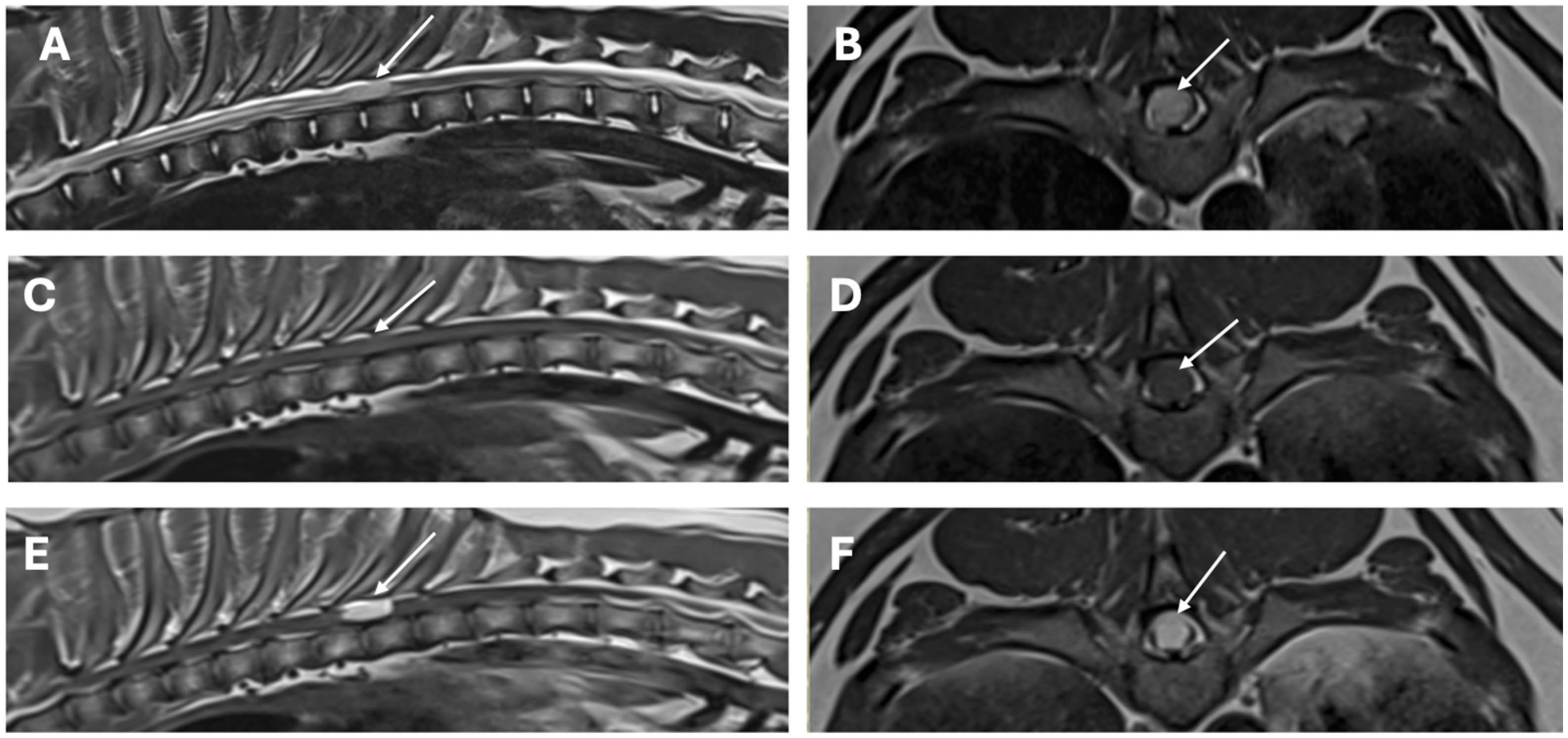
MRI study of the thoracolumbar region. Sagittal images [**A** (T2-weighted), **C** (T1-weighted), **E** (T1-weighted post-contrast)] and transverse images [**B** (T2-weighted), **D** (T1-weighted), and **F** (T1-weighted post-contrast)] at the level of the lesion. There is a single, well-defined, intramedullary T2-weighted hyperintense, T1-weighted isointense with homogenous strong contrast uptake lesion (arrows), affecting almost 90% of the diameter of the spinal cord at the level of T8–T9 vertebrae.

**Figure 2:**
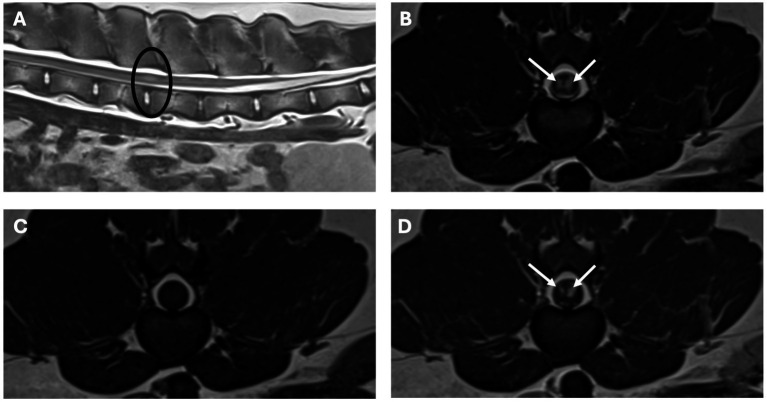
MRI study of the lumbar region. Sagittal image [**A** (T2-weighted) and transverse images, **B** (T2-weighted), **C** (T1-weighted), **D** (T1-weighted post-contrast)]. The lesion is circled in the sagittal image, in the transverse images there are bilateral lesions, well-defined, intramedullary T2-weighted hyperintense, T1-weighted isointense with homogenous strong contrast uptake (arrows), affecting mainly the grey matter, more marked on the left side at the level of L3–L4 vertebrae.

MRI of the thoracolumbar spine (Figures 1, 2) revealed two intramedullary lesions, one located from the caudal aspect of T8 to the mid aspect of T9 vertebral level, and a second one located at the level of L3–4 vertebrae. The intramedullary lesion within the thoracic area was well-defined, oval, and occupied most of the diameter of the spinal cord. It was homogeneous, hyperintense in T2W, isointense in T1W, and it showed severe and homogeneous contrast enhancement. The second lesion at the L3–L4 vertebrae affected mainly the grey matter, more marked on the left side, and it showed the same signal intensity pattern. There was moderate dilation of the central canal at the level of C7 to T7 vertebrae. Considering the multifocal pattern of the lesions, MRI of the remaining vertebral column and brain was also performed, which appeared unremarkable.

The main differential diagnosis for multiple intramedullary lesions included neoplasia, such as lymphoma or glioma. The second lesion affecting grey matter could also be compatible with an ischemic myelopathy, which could also be secondary to neoplasia.

Serum biochemistry revealed an increased creatinine kinase 1,985 U/L (reference range 20–230), and hematology was unremarkable. Cerebrospinal fluid (CSF) analysis obtained via lumbar puncture revealed mild mononuclear pleocytosis with a total nucleated cell count of 6 cells/μL (normal <5 cells/μL) and increased protein of 214.9 mg/dL (normal <45 mg/dL). *Neospora caninum* antibodies serum levels and *Cryptococcus* antigen tests were negative.

Initially medical management was attempted with meloxicam 0.1 mg/kg SID PO and clindamycin 12.5 mg/kg BID PO, whilst awaiting laboratory results. The dog’s clinical signs progressed, and the dog became non-ambulatory paraparetic within 4 days.

Exploratory surgery for biopsy or possible resection of the thoracolumbar mass was offered. Surgery was performed by means of a right-sided T7–T9 hemilaminectomy and durotomy. The spinal cord was visibly enlarged in the region corresponding to the mass. Following durotomy, the mass had a mildly harder consistency than spinal parenchyma and adhered to normal parenchyma, particularly in its ventral portion. There was no apparent connection of the mass to the dura mater. The mass was focal, cauliflower-shaped, white colored, and not dissimilar but slightly darker than the normal spinal cord parenchyma. The majority of the affected tissue evident macroscopically, was removed, although it was strongly adhered to the spinal cord parenchyma, and resection was only possible by sharp dissection. The obtained tissue samples were sent for histopathological analysis. The dog recovered uneventfully from anesthesia and remained hospitalized, having received methadone (0.3 mg/kg) IV q4h, gabapentin 11 mg/kg PO q8h, and methylprednisolone 0.1 mg/kg IV. Following surgery, the patient became paraplegic without nociception.

Initial histopathology of the T7–T9 mass lesion revealed the presence of a markedly infiltrative malignant proliferation, suggesting neoplasia, consisting mainly of spindle cells, effacing a significant portion of the spinal cord, although the cell of origin remained uncertain. Following 4 days of hospitalization, considering the initial histopathology findings indicating a poor prognosis, the owner opted for euthanasia, with a necropsy being performed.

**Figure 3:**
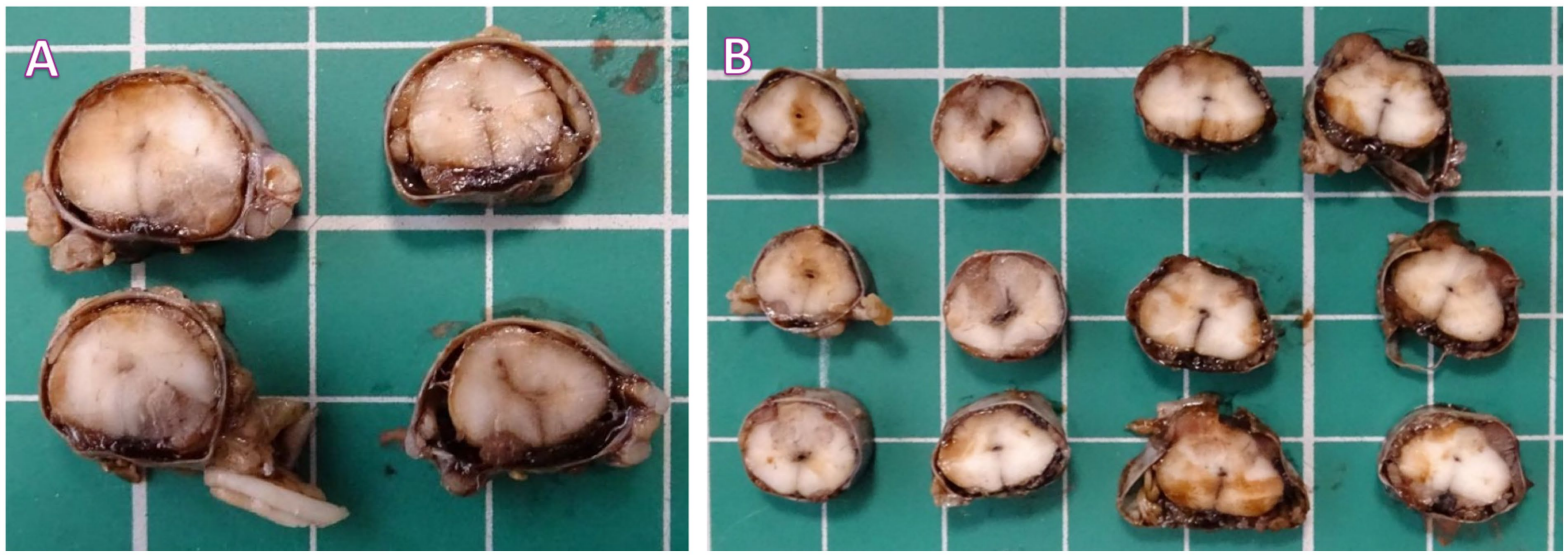
**(A)** Cervicothoracic intumescence. Multifocal grey discolored areas arising within the subdural space and variably infiltrating and distorting the neuroparenchyma. **(B)** Thoracic and lumbar spinal cord. Multifocal grey discolored areas arising within the subdural space/nerve roots and variably infiltrating and distorting the neuroparenchyma.

The brain and entire spinal cord were collected at necropsy and trimmed after fixation in 10% buffered formalin. On macroscopic examination, at the level of the cervicothoracic intumescence, thoracic and lumbar spinal cord, there were multifocal, grey discolored areas which appear to arise within the subdural space and from the nerve roots and multifocally, variably infiltrate and distort the spinal neuroparenchyma, accompanied by multifocal subdural hemorrhage (Figure 3).

**Figure 4:**
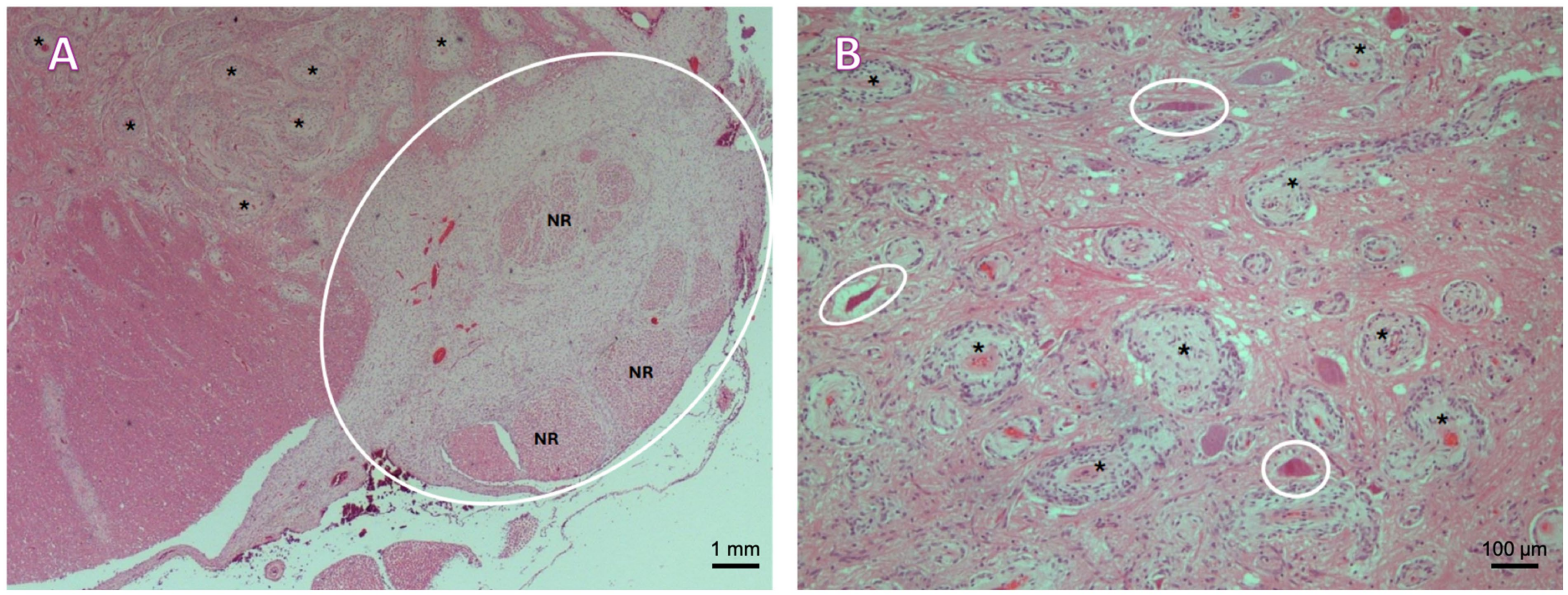
**(A)** Lumbar spinal cord. A plaque-like proliferation of spindle cells arranged in bundles and whorls around numerous, proliferating small to medium caliber vessels supported by variable amounts of loosely arranged fibrillar stroma is observed within the ventral leptomeninges (cycled), entrapping and expanding multiple nerve roots (NR), and multifocally infiltrating and expanding the Virchow–Robin spaces within the adjacent ventral funiculi and spinal grey matter (asterisks). H&E, 2.5×. **(B)** Cervicothoracic intumescence, grey matter. Multifocal proliferation of spindle-shaped to irregularly polygonal cells arranged in bundles and whorls around numerous, proliferating small to medium caliber vessels (asterisks). Multifocal necrotic neurons within the affected area (circled). H&E, 10×.

Histopathology of the whole spinal cord revealed prominent pathological changes involving various segments of the spinal cord (Figure 4). The lesions consisted of multifocal to coalescing proliferations of leptomeningeal and meningovascular structures, which variably effaced the white and grey matter of the cervicothoracic, thoracic, thoracolumbar, and lumbar segments of the spinal cord. Based on the histopathological findings, the lesions were consistent with multifocal to coalescing meningioangiomatosis. Gross and histopathological examination of the brain was unremarkable.

## 3 Discussion

Several instances of proliferative vascular disorders (considered malformations, benign neoplasms, or hamartomas) directly or indirectly affecting the CNS in dogs have been reported, including arteriovenous malformation, cavernous hemangioma (angioma), capillary telangiectasis, venous angioma, MA, and angiomatosis (1). Angiomatosis is an unusual disorder of blood vessels (1), and several forms have been reported in dogs: cutaneous (17), meningeal (3), multi-systemic (18), and skeletal (19, 20). The etiology of MA is unclear, as it could represent a hamartoma, a form of leptomeningeal meningioma, or a vascular malformation (6, 21).

In human medicine, MA can present in two forms: sporadic and neurofibromatosis type 2 (NF2) associated forms. While the sporadic form is associated with seizures and headaches, the NF2-associated form is normally asymptomatic (21). In human MA, all cases have been reported to affect the brain, with no instance of spinal cord involvement. Common histological findings in humans include cortical meningovascular proliferation with spindle-shaped cells infiltration along the Virchow–Robin canal, calcification, psammoma bodies, fibrosis, and white matter infiltration (22). The sporadic form of human MA has been reported concomitantly with other CNS lesions such as meningioma (21, 22). In dogs, there has been a single case report of MA and fibrous meningioma in a dog (10) and one case of MA with glioma (13).

A total of 15 cases of MA have been previously reported in dogs (1, 3–13). Affected breeds included Labrador Retrievers (4/15) (8, 10–12), crossbreed (2/15) (5, 7), German Shepherd (2/15) (3, 9), and a single instance of Australian Cattle Dog (7), Australian Shepherd (7), Boxer (8), Campeiro Bulldog (13), and Portuguese Water Dog (7). Although frequently only young dogs were reported as affected (7), a considerable age range has been reported since, including cases ranging from 3 months up to 14 years of age (1, 3–13). Neurological presentation in dogs will depend on lesion localization, with vestibular ataxia, progressive gait abnormalities, ventral flexion of the neck, progressive brainstem signs, and seizures described in intracranial cases (4, 7, 10). The vast majority of canine MA cases have been reported affecting the brain (12/15), with the brainstem being the most common anatomical region affected (7/15), with lesions more specifically affecting the mesencephalon, metencephalon, and prosencephalon (1, 3–13). In our case, the brain was unremarkable on MRI, as well as macroscopically on necropsy examination and histologically.

In our case, multiple mass-like spinal cord lesions compatible with MA have been found involving various segments of the spinal cord. Three previous case reports described single cervical or thoracolumbar MA in dogs. A summary of the literature describing cases with a single site spinal cord lesion can be found on Table 1 (7, 8, 11).

**Table 1:** Table summarising findings of previous case reports of meningioangiomatosis in dogs (MA).

Case	Signalment	Clinical features	MRI findings	Treatment and outcome	Histopathological findings
Cervical MA case (7)	14-month-old spayed female Australian Cattle Dog	Chronic progressive spastic tetraparesis	Cervical myelography and CT: mild attenuation of dorsal and ventral contrast columns from C2 to C6; no definitive spinal cord lesion identified	Euthanasia without attempted treatment	Thin meningeal plaque from C4 to C6; mild central canal dilation; demyelination of dorsolateral and lateral funiculi; multifocally pale ventral funiculi caudally; brain and remaining spinal cord macroscopically normal
Thoracolumbar MA case (8)	4-year-old male Boxer	Chronic progressive paraparesis and fecal incontinence	MRI: T2W hyperintense lesion with hypointense center, FLAIR hyperintense, mild T1W and GRE hyperintensity, homogeneous contrast enhancement at T13	Euthanasia without attempted treatment	Wedge-shaped lesion at cranial T13 spinal cord; infiltration from leptomeninges into spinal cord parenchyma; no other spinal cord sections described
Thoracolumbar MA case (11)	5-year-old neutered male Labrador Retriever	8-week history of progressive abnormal pelvic limb gait; T3–L3 spinal cord localization	CT myelogram: expansile mass with intramedullary filling at L1; MRI: circumscribed, markedly T2W/STIR hyperintense, mild T1W hyperintense mass with peripheral contrast enhancement	Surgical debulking via hemilaminectomy and durotomy; follow-up 18 months with no deterioration	Easily removed mass lesion; presumed intradural (possibly intramedullary) origin

CT, computed tomography; FLAIR, fluid-attenuated inversion recovery; GRE, gradient echo imaging; MRI, magnetic resonance imaging; STIR, Short Tau Inversion Recovery; T1W, T1-weighted imaging; T2W, T2-weighted imaging.

In our case, in line with the previous literature, a young Labrador Retriever presented with chronic progressive T3–L3 myelopathy. MRI of the thoracolumbar spine revealed two presumed intramedullary lesions. The lesions were both homogeneous, T2W hyperintense, T1W isointense, with severe and homogeneous contrast enhancement. In humans, where MRI is frequently the imaging modality of choice, the most commonly observed MRI features are T1 hypo- to iso-intensity along with T2 and FLAIR (fluid attenuated inversion recovery) hyperintensity and variable contrast enhancement (2). In people, CT can reveal hypodensity lesions with possible contrast-enhancement, besides evidence of calcification, which can develop as fibrocalcifying changes and even osseous metaplasia takes place in advanced cases (2). Nonetheless, diagnostic imaging features of MA are variable and not considered specific in both people and dogs, making preoperative diagnosis challenging or impossible (2, 8). As an intradural intramedullary lesion, the main differential diagnosis in dogs would typically include glioma, ependymoma, nephroblastoma (depending on location), nerve sheath tumors (if associated with the nerve root), or vascular malformations, such as MA, or metastatic neoplasia (23). Considering the leptomeningeal involvement in MA cases, meningeal involvement would be expected; however, both our cases and the other cases with MRI revealed an intramedullary pattern (8, 11). Furthermore, in our case, several other lesions evident on histopathology involving the cervicothoracic and thoracic spinal cord were not visible on MRI. Negative MRI findings with MA are reported in the human literature, and cases typically affect the grey matter, like in our case (24).

Cisternal CSF analysis was unremarkable in previous reports in dogs (7, 12), except for a case where MA was described in association with a fibrous meningioma in the brain of a dog, with neutrophilic pleocytosis (166 cells/μL, reference range <5 cells/μL) and an elevated total protein content (35 mg/dL, reference range <25 mg/dL) (10). In our case, lumbar CSF obtained revealed mild mononuclear (6 cells/μL, reference <5 cells/μL) and increased total protein content (214.9 mg/dL, lumbar reference range <45 mg/dL). This suggests that CSF findings in spinal MA cases might be non-specific. Histological findings characterizing MA within the spinal cord in previously reported canine cases consisted of variably spindle, meningothelial cells, and vascular proliferations with a low mitotic index, similar to what was observed in our case (8, 11).

Treatment of MA can be challenging as the fundamental pathophysiology of these lesions remains unknown. The treatment of choice for MA in human medicine is surgical resection when faced with an unifocal lesion; however, in cases of diffuse or multifocal MA, surgical resection is not possible (2). Another line of treatment described in human medicine is antiangiogenic therapy with bevacizumab, which prevents angiogenesis due to interference with endothelial growth factor (25). In veterinary medicine, unsuccessful medical management with antibiotics and corticosteroids has been described (7, 12). Surgical management was reported to lead to a good prognosis in a single case, when surgical resection was achievable (11). Unfortunately, this was not possible in our case, due to the multifocal and infiltrative pattern of the proliferation and to the intraoperative finding of a well-defined mass that was strongly adherent to the spinal cord parenchyma, such that resection was only possible using sharp dissection (11).

Our case report highlights the variable and complex nature of MA. Of the several infiltrative lesions found on necropsy, only two were identifiable on high-field MRI, and surgical treatment was not successful, contrary to a single previous account. This is the first case report of multifocal spinal MA in the veterinary literature. Imaging features described in this case report can be useful, similar to previous reports of spinal MA; however, still not necessarily specific. Our findings highlight MA as a further possible differential diagnosis for multifocal intramedullary masses affecting young dogs.

## Data Availability

The data analyzed in this study is subject to the following licenses/restrictions: owner provided permission for using data for teaching and research. Requests to access these datasets should be directed to sergio.gomes@dovecotereferrals.co.uk.
